# Analysis of Wearable and Smartphone-Based Technologies for the Measurement of Barbell Velocity in Different Resistance Training Exercises

**DOI:** 10.3389/fphys.2017.00649

**Published:** 2017-08-28

**Authors:** Carlos Balsalobre-Fernández, David Marchante, Eneko Baz-Valle, Iván Alonso-Molero, Sergio L. Jiménez, Mario Muñóz-López

**Affiliations:** ^1^School of Sports Science, European University of Madrid Madrid, Spain; ^2^Department of Research and Development, PowerExplosive Center Madrid, Spain

**Keywords:** monitoring, biomechanics, technology, strength, validation

## Abstract

The purpose of this study was to analyze the validity, reliability, and accuracy of new wearable and smartphone-based technology for the measurement of barbell velocity in resistance training exercises. To do this, 10 highly trained powerlifters (age = 26.1 ± 3.9 years) performed 11 repetitions with loads ranging 50–100% of the 1-Repetition maximum in the bench-press, full-squat, and hip-thrust exercises while barbell velocity was simultaneously measured using a linear transducer (LT), two *Beast* wearable devices (one placed on the subjects' wrist –BW–, and the other one directly attached to the barbell –BB–) and the iOS *PowerLift* app. Results showed a high correlation between the LT and BW (*r* = 0.94–0.98, SEE = 0.04–0.07 m•s^−1^), BB (*r* = 0.97–0.98, SEE = 0.04–0.05 m•s^−1^), and the *PowerLift* app (*r* = 0.97–0.98, SEE = 0.03–0.05 m•s^−1^) for the measurement of barbell velocity in the three exercises. Paired samples *T*-test revealed systematic biases between the LT and BW, BB and the app in the hip-thrust, between the LT and BW in the full-squat and between the LT and BB in the bench-press exercise (*p* < 0.001). Moreover, the analysis of the linear regression on the Bland-Altman plots showed that the differences between the LT and BW (*R*^2^ = 0.004–0.03), BB (*R*^2^ = 0.007–0.01), and the app (*R*^2^ = 0.001–0.03) were similar across the whole range of velocities analyzed. Finally, the reliability of the BW (ICC = 0.910–0.988), BB (ICC = 0.922–0.990), and the app (ICC = 0.928–0.989) for the measurement of the two repetitions performed with each load were almost the same than that observed with the LT (ICC = 0.937–0.990). Both the *Beast* wearable device and the *PowerLift* app were highly valid, reliable, and accurate for the measurement of barbell velocity in the bench-press, full-squat, and hip-thrust exercises. These results could have potential practical applications for strength and conditioning coaches who wish to measure barbell velocity during resistance training.

## Introduction

Quantifying and monitoring intensity is a key part when designing resistance training programs (Folland and Williams, 2007; Borresen and Lambert, 2009; Tillin and Folland, 2014); in fact, training intensity is considered the most important variable to produce the desired neuromuscular adaptations (Folland and Williams, 2007). Several approaches have been used for decades to monitor the intensity during resistance training (Gonzalez-Badillo and Sánchez-Medina, 2010; Zourdos et al., 2015; Naclerio and Larumbe-Zabala, 2016), with the 1-Repetition maximum (1-RM) being the most widely used in the field of strength and conditioning (Buckner et al., 2017). To measure the 1-RM, the athlete needs to perform a maximal lift with a load that can be moved just once; therefore, given the extreme effort it represents, different strategies have emerged to indirectly estimate the 1-RM in a less demanding way (Kravitz et al., 2003; Robertson et al., 2008; Jidovtseff et al., 2011; Picerno et al., 2016). Among them, the measurement of the velocity at which the barbell is moved in the concentric phase on different resistance exercises has been shown to provide accurate, indirect estimations of the 1-RM without the need to perform a maximal lift (Gonzalez-Badillo and Sánchez-Medina, 2010; Jidovtseff et al., 2011; Picerno et al., 2016; Muñoz-López et al., 2017). These studies, mainly conducted within the last decade, are based on the very high correlation (*R*^2^ > 0.97) observed between the load (in terms of %1-RM) and the mean velocity at which each load is lifted (Gonzalez-Badillo and Sánchez-Medina, 2010; Conceição et al., 2016; Muñoz-López et al., 2017). Thus, based on load-velocity profiles, the measurement of movement velocity during resistance training can be used to estimate 1-RM and each of its percentages, which could help adjusting training intensity (Muñoz-López et al., 2017). Moreover, movement velocity during resistance training has shown to provide accurate estimations of the degree of neuromuscular fatigue (Sánchez-Medina and Gonzalez-Badillo, 2011). For example, it has been observed that manipuling the percentage of velocity loss during the set influences the increases in strength and hypertrophy (Pareja-Blanco et al., 2016), with lower percentages of loss being associated with greater performance improvements and less muscle mass gains. Therefore, analyzing the drop of velocity within the sets could be used to optimize the adaptations to resistance training (Sánchez-Medina and Gonzalez-Badillo, 2011; Tufano et al., 2016).

To measure barbell velocity, different technologies, such as accelerometers, professional video systems or linear transducers (LT) have been used, with LT often considered the gold standard (Balsalobre-Fernández et al., 2016b, 2017; Banyard et al., 2016). An LT consists of a sensor with a cable that is attached to the barbell and measures barbell velocity by differentiating cable displacement with respect to time. However, many available LTs are still too expensive for many coaches (~US$2,000), which prevents its use outside professional clubs or sports sciences laboratories. For this reason, recent studies have analyzed the validity and reliability of more affordable technologies used to measure barbell velocity in resistance exercises, like high-speed cameras, smartphone apps, or wearable devices (Balsalobre-Fernández et al., 2016b, 2017; Sañudo et al., 2016). For example, an iOS app named *PowerLift* was recently validated for the measurement of barbell velocity with respect to a 1 kHz LT in the bench-press exercise (Balsalobre-Fernández et al., 2017). Specifically, the use of wearable devices in sports sciences is getting a lot of attention during the last years; in fact, “Wearable devices” is the #1 fitness trend in the 2017 edition of the American College of Sports Medicine survey (Thompson, 2016). However, the use of wearable devices for the measurement of physical performance is currently questioned (Halson, 2016), since just a few of the hundreds of models available are scientifically validated. For example, to the best of our knowledge, there is just one validated wearable device for the measurement of barbell velocity in resistance exercises (i.e., the PUSH band) (Balsalobre-Fernández et al., 2016b; Banyard et al., 2017). Thus, the purpose of the present study was to analyze the concurrent validity and reliability of a popular wearable device (i.e., the *Beast* sensor) for the measurement of barbell velocity in the full-squat, bench-press, and hip-thrust exercises. Additionally, we aimed to test if the positive results observed in the previously validation paper of the *PowerLift* iOS app (Balsalobre-Fernández et al., 2017) were confirmed in the full-squat and the hip-thrust exercises, since movement velocity can greatly vary between different exercises (Sánchez-Medina et al., 2014; Conceição et al., 2016).

## Materials and methods

### Participants

Ten highly-trained, competitive powerlifters at national and international events took part in this study [*N* = 10; 6 men, 4 women; age = 26.1 ± 3.9 years, body mass index = 23.2 ± 3.3 kg/m^2^, 1-RM relative to body mass (kg/kg): 1.93 ± 0.5 –full-squat–, 1.3 ± 0.5 –bench-press–, 2.9 ± 0.7 –hip-thrust–]. The study protocol complied with the Declaration of Helsinki for Human Experimentation and was approved by the ethics committee at the European University of Madrid, Spain. Written informed consent was obtained from each participant in advance.

### Experimental design

Participants performed six incremental sets until they reached their 1-Repetition maximum on the full-squat, bench-press, and hip-thrust exercises while mean barbell displacement velocity was measured with a Smartcoach Power Encoder linear transducer (LT), two *Beast sensor* wearable devices (one fixed to the barbell –BB–, another one attached to a wrist-band that the participants wore on their right wrist –BW–) and the *PowerLift* iOS app simultaneously. Two repetitions were completed with the five initial sets (which corresponded approximately to 50, 60, 70, 80, and 90% of the 1-RM), while one repetition was performed with the last set (i.e., the 100% 1-RM); therefore, 11 repetitions were measured for each participant and exercise for a total of 330 repetitions. Several statistical analyses were conducted in order to compare the velocities measured with BB, BW and the app with those obtained with the LT. Finally, the load-velocity profiles (i.e., the slope, y-intercept and coefficient of determination of the regression line) computed with the loads used in the six incremental sets (in kg) and its associated velocities measured with each device were compared.

### 1-RM incremental test

Participants were asked to perform three 1RM tests, one for each exercise (i.e., full squat, bench press, and hip-thrust), on three separate occasions interspersed with 48 h of passive rest. The 1-RM incremental tests consisted of five sets with loads ranging ~50–90% 1-RM, and one last set with the actual 1-RM. With the submaximal loads, participants performed two repetitions, while with the 1-RM they did just one. When participants were able to perform more than one repetition, an additional set with a heavier load was performed in order to reach the 1-RM. Sets were separated by 5 min of passive rest. Athletes were instructed to perform each repetition as fast as possible, and all of them were experts on the full-squat, bench-press, and hip-thrust exercises.

### Data collection

#### Linear transducer

**Figure 1:**
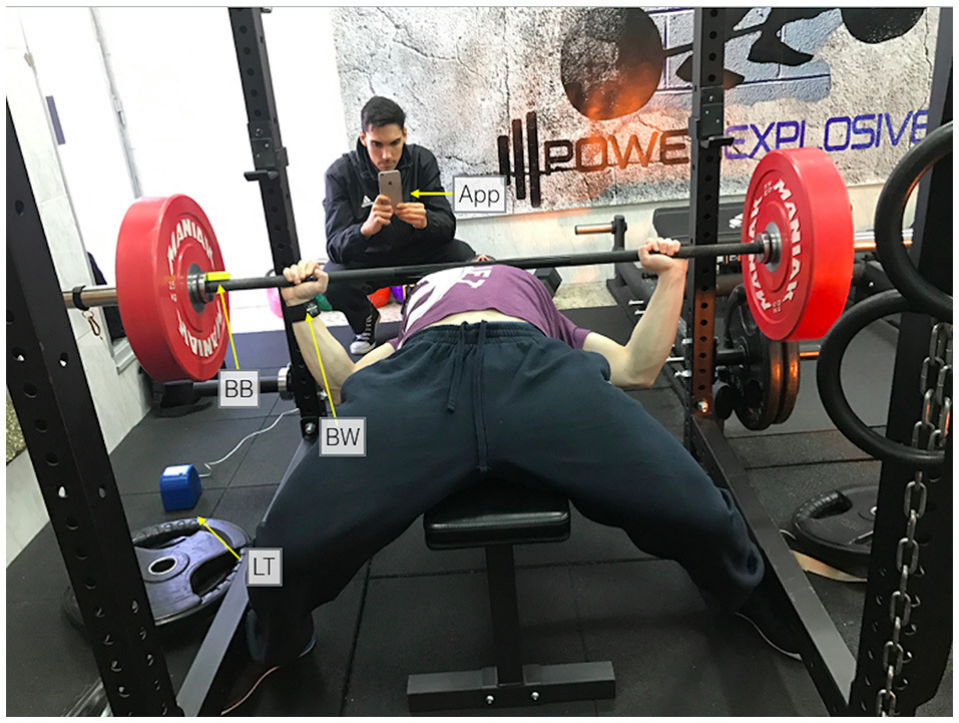
Setup of the different devices during the measurement of barbell velocity in the bench-press exercise. App: *PowerLift* app; BW = *Beast* sensor (wrist); BB = *Beast* sensor (barbell); LT = linear transducer. Written informed consent was obtained from the two identifiable subjects for the publication of this picture.

A *SmartCoach Power Encoder* (SmartCoach Europe, Stockholm, Sweden) linear transducer (LT) was considered as the criterion for the measurement of barbell displacement velocity in the present study. Mean velocity (in m•s^−1^) of each repetition was recorded at a sampling rate of 1 kHz by attaching the cable of the LT to the barbell, aligned with the vertical axis as described by the manufacturer (i.e., perpendicular to the ground). Specifically, the cable was attached to the right end of the barbell, close to the weight plates. See Figure 1. Then, the LT was connected to the Smartcoach software 5.0.0 installed on a personal computer running the Windows 10 operating system, which provided mean velocity values in real time.

#### Beast sensor wearable device

The *Beast sensor* is a small device (15.2 cm^3^ of volume, 38 g of weight) which includes a 3-axis accelerometer, gyroscope and magnetometer that measures velocity at a sampling rate of 50 Hz. This wearable was designed to be fixed to a wristband that the athlete wears while training. Also, it can be positioned on to the barbell thanks to a built-in magnet. In order to test both configurations, two *Beast sensors* were used in the present study: one was directly fixed to the right end of the barbell (BB) and another one was placed in the right wrist of the participants using a wristband designed by the same manufacturer (BW). See Figure 1 for more details. Then, mean velocity (in m•s^−1^) of each repetition was transferred in real time via Bluetooth 4.0 LE to the Beast app for iOS v.2.2.3, which was installed on an iPhone 6 with iOS 10.2.1 operative system.

#### PowerLift app

Finally, the *PowerLift v.4.0* iOS app, installed on an iPhone 6 running iOS 10.2.1 was used to measure barbell velocity in the three mentioned exercises as well. The app was designed to measure barbell velocity by video-recording the lift at slow motion thanks to the high-speed camera included in current iOS devices. Then, the app allowed a frame-by-frame inspection of the video to manually select the beginning and end of the movement, and therefore measure the time of the concentric phase of the lift. Finally, mean vertical barbell velocity (in m•s^−1^) was computed as the range of motion (ROM) of the concentric phase of the exercise divided by the time of the lift. For the full-squat exercise, the ROM was calculated as the vertical difference between the height of the barbell with respect to the ground on the final position (i.e., knees extended) and the height of the barbell at the bottom position (i.e., thighs parallel to the ground). The beginning of the movement was considered as the first frame in which the barbell started to ascend vertically, while the end was considered as the first frame in which the barbell stopped that ascension. For the bench-press exercise, the ROM was calculated as the vertical distance between the barbell in the final position of the exercise (i.e., elbows fully extended) and the chest of the athlete. The beginning of the lift was considered as the first frame in which the barbell left the chest of the participant, while the end was considered as the first frame in which the barbell ended its vertical displacement. Finally, in the hip-thrust exercise, the ROM was calculated as the vertical distance between the bottom of the plate in the final position of the exercise (i.e., hip extended, thighs parallel to the ground) and the ground. The beginning of the movement was considered as the first frame in which the weight plates left the ground, while the end was considered as the first frame in which the vertical displacement of the barbell ends. To measure the ROM, an experienced researcher used a metric tape, while beginning and end frames were manually selected by an observer with experience in video analysis. All the videos were recorded and analyzed at 240 frames per second (FPS), at a quality of 720 p.

### Statistical analyses

Several statistical analyses were used to test the accuracy, validity, and reliability of the *Beast* wearable device and the *PowerLift* app for the measurement of barbell velocity in the full-squat, bench-press and hip-thrust exercises in comparison with the gold standard (i.e., the LT).

### Accuracy

Paired samples *t*-test and Bland–Altman plots were used to identify potential systematic bias, reported via mean absolute (in m/s) and relative (in %) bias and standard deviations. Furthermore, standard error of estimate (SEE) was also used to report the typical error in the measurements in comparison with the LT.

### Validity

Concurrent validity was tested using Pearson's product–moment correlation coefficient (r) with 90% confidence intervals (CI) via *N* = 1,000 bootstrapping. Scores from 0.8 to 0.9 were considered as good, while values above > 0.9 were considered as high (Vincent and Weir, 2012). Moreover, the analysis of the regression line on the Bland–Altman plots was used to check for heterogeneity of the observed differences across the whole range of velocities analyzed. Finally, one way ANOVA was used to compare the load-velocity profiles between the four devices analyzed (i.e., the LT, BW, BB, and the *PowerLift* app).

### Reliability

To test the reliability of the different devices for the measurement of the two repetitions performed on the 5 initial sets of the incremental test, the intraclass correlation coefficient (ICC) with 90% CI was used. Scores from 0.8 to 0.9 were considered as good, while values above > 0.9 were considered as high (Vincent and Weir, 2012).

The level of significance was set at 0.01. All calculations were performed using IBM® SPSS® Statistics 22 software (IBM Co., USA).

## Results

### Validity and accuracy

**Figure 2:**
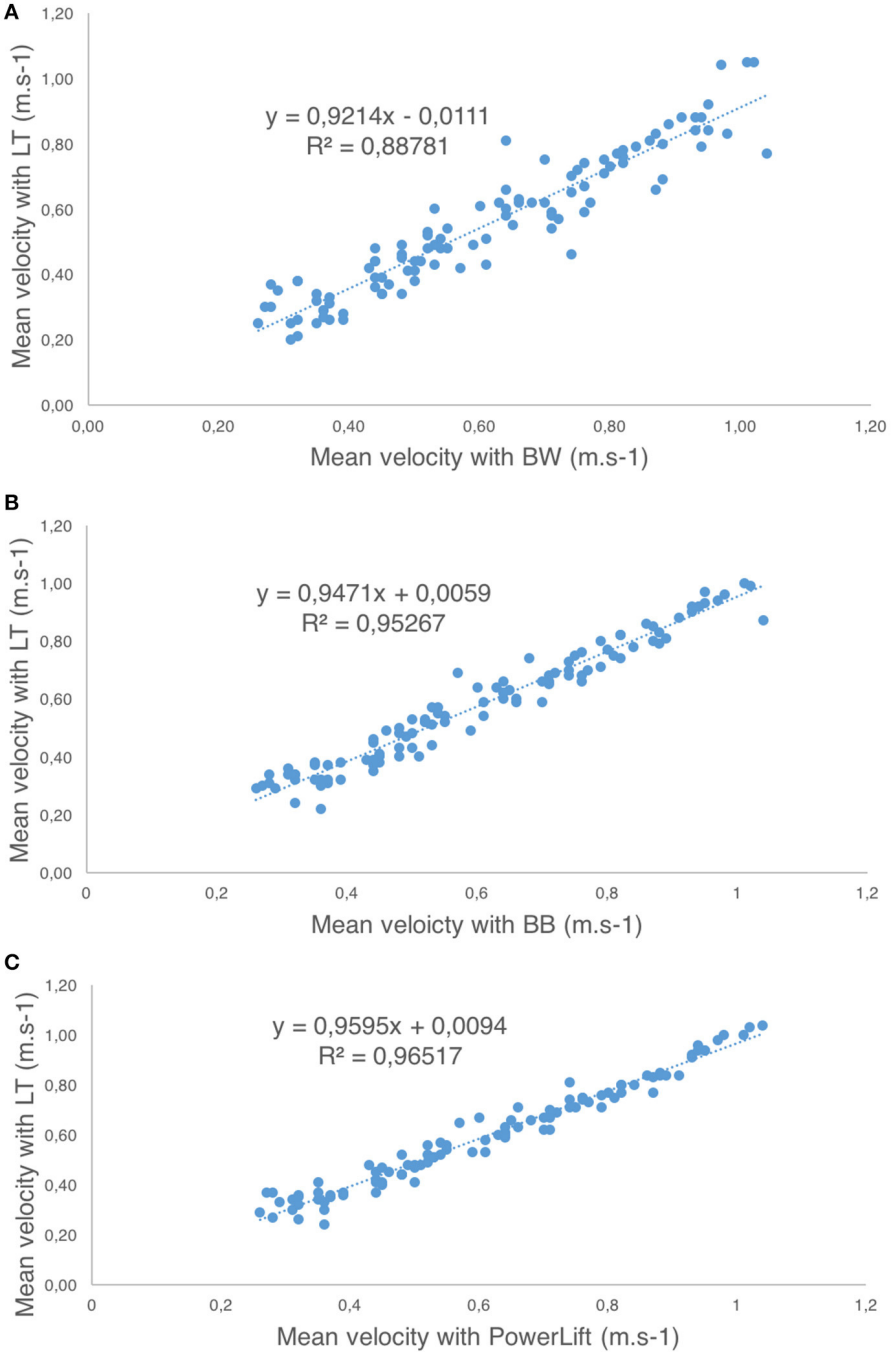
Correlation with first order regression line between the linear transducer (LT) and: **(A)** *Beast* sensor (wrist, BW); **(B)** *Beast* sensor (barbell, BB); **(C)** *PowerLift* app for the hip-thrust exercise.

Pearson's product-moment correlation coefficient revealed a high correlation between the values of mean velocity measured with the linear transducer and BW (*r* = 0.94–0.98, SEE = 0.04–0.07 m•s^−1^), BB (*r* = 0.97–0.98, SEE = 0.04–0.05 m•s^−1^), and the *PowerLift* app (*r* = 0.97–0.98, SEE = 0.03–0.05 m•s^−1^) for the three exercises analyzed. See Figure 2.

**Figure 3:**
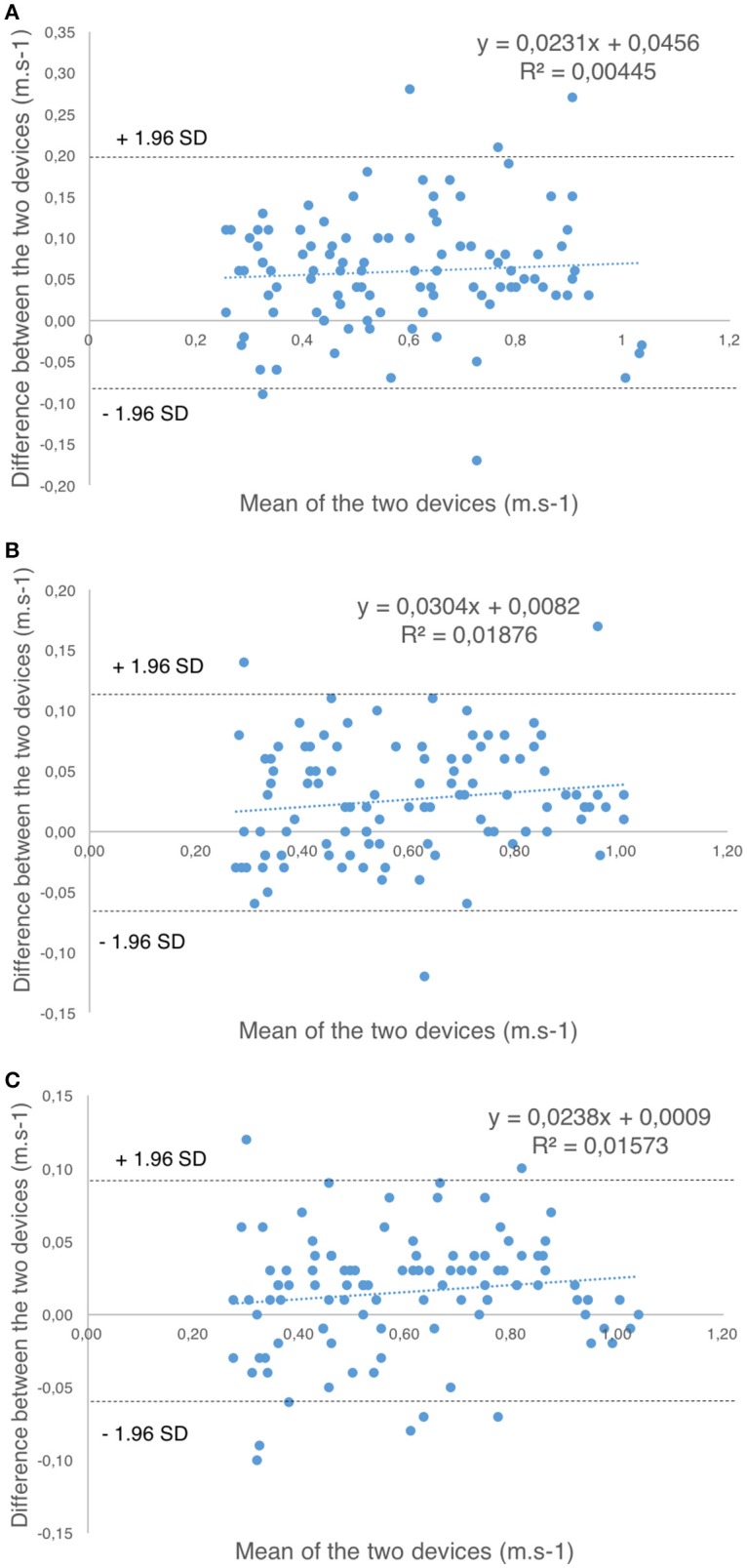
Bland-Altman plots for the measurement of barbell velocity between the linear transducer (LT) and: **(A)** *Beast* sensor (wrist, BW); **(B)** *Beast* sensor (barbell, BB); **(C)** *PowerLift* app for the hip-thrust exercise. The blue dashed line represents the first-order regression line of the data, while the gray dashed lines represents ±1.96 standard deviations (*SD*).

When analyzing the accuracy of the *Beast sensor* and the *PowerLift* app for the measurement of barbell velocity in comparison with the LT, systematic biases where observed for BW, BB, and the app in the hip-thrust and the bench-press exercise, and for BW in the full-squat exercise (*p* < 0.001), with values of the LT being systematically higher in most cases [BW (mean difference = 0.01–0.06 m•s^−1^); BB (mean difference = 0.03–0.04 m•s^−1^); *PowerLift* app (mean difference = −0.01 to 0.02 m•s^−1^)]. See Table 1 for more details. Moreover, differences between the LT and each device were similar across the whole range of velocities analyzed as revealed by the linear regression of the Bland-Altman plots (BW: *R*^2^ = 0.004–0.03, BB: *R*^2^ = 0.007–0.01, *PowerLift: R*^2^ = 0.001–0.03). See Figure 3.

**Table 1:** Concurrent validity of the three devices analyzed for the measurement of barbell velocity in comparison with a linear transducer.

	**Full-squat**				**Bench-press**				**Hip-thrust**			
	**Pearson's *r***	**Absolute bias (m•s^−1^)**	**Relative bias (%)**	**SEE (m•s^−1^)**	**Pearson's *r***	**Absolute bias (m•s^−1^)**	**Relative bias (%)**	**SEE (m•s^−1^)**	**Pearson's *r***	**Absolute bias (m•s^−1^)**	**Relative bias (%)**	**SEE (m•s^−1^)**
Beast sensor (wrist)	0.971 (0.960–0.979)	0.03 ± 0.06^*^	15.6 ± 13.2	0.06	0.983 (0.976–0.988)	0.009 ± 0.04	24.2 ± 12.6	0.04	0.942 (0.919–0.961)	0.06 ± 0.07^*^	14.4 ± 10.9	0.07
Beast sensor (barbell)	0.980 (0.973–0.983)	–0.003 ± 0.05	8.8 ± 7.9	0.05	0.978 (0.968–0.985)	0.04 ± 0.05^*^	11.6 ± 15.2	0.05	0.976 (0.967–0.984)	0.03 ± 0.05^*^	8.0 ± 7.4	0.04
PowerLift app	0.986 (0.979–0.991)	–0.005 ± 0.04	8.4 ± 6.5	0.04	0.973 (0.959–0.984)	–0.01 ± 0.05	10.0 ± 10.2	0.05	0.982 (0.975–0.988)	0.02 ± 0.04^*^	6.7 ± 6.4	0.03

*Pearson's r = Pearson's product-moment correlation coefficient. SEE = standard error of the estimate. Values between brackets represents 90% confidence intervals computed via N = 1,000 bootstrapping*.

* *p < 0.05*.

### Reliability

When analyzing the two repetitions performed with each load ranging 50–95% 1-RM, high agreements were observed for all the devices and exercises analyzed (ICC > 0.9). Moreover, the levels of reliability of the BW, BW, and the *PowerLift* app were very similar to those observed with the LT. See Table 2 for more details.

**Table 2:** Intraclass correlation coefficient for the measurement of the two repetitions performed with loads ranging 50–95% 1-RM with the three exercises, for all the devices analyzed.

	**Full-squat**	**Bench-press**	**Hip-thrust**
Linear transducer	0.981 (0.965–0.990)	0.981 (0.965–0.990)	0.966 (0.937–0.982)
Beast sensor (wrist)	0.975 (0.955–0.986)	0.977 (0.958–0.988)	0.952 (0.910–0.974)
Beast sensor (barbell)	0.979 (0.962–0.988)	0.981 (0.966–0.990)	0.958 (0.922–0.977)
PowerLift app	0.981 (0.965–0.989)	0.974 (0.951–0.986)	0.961 (0.928–0.979)

*ICC = Intraclass correlation coefficient. Values between brackets represents 90% confidence intervals computed via N = 1,000 bootstrapping*.

### Load-velocity profile

**Figure 4:**
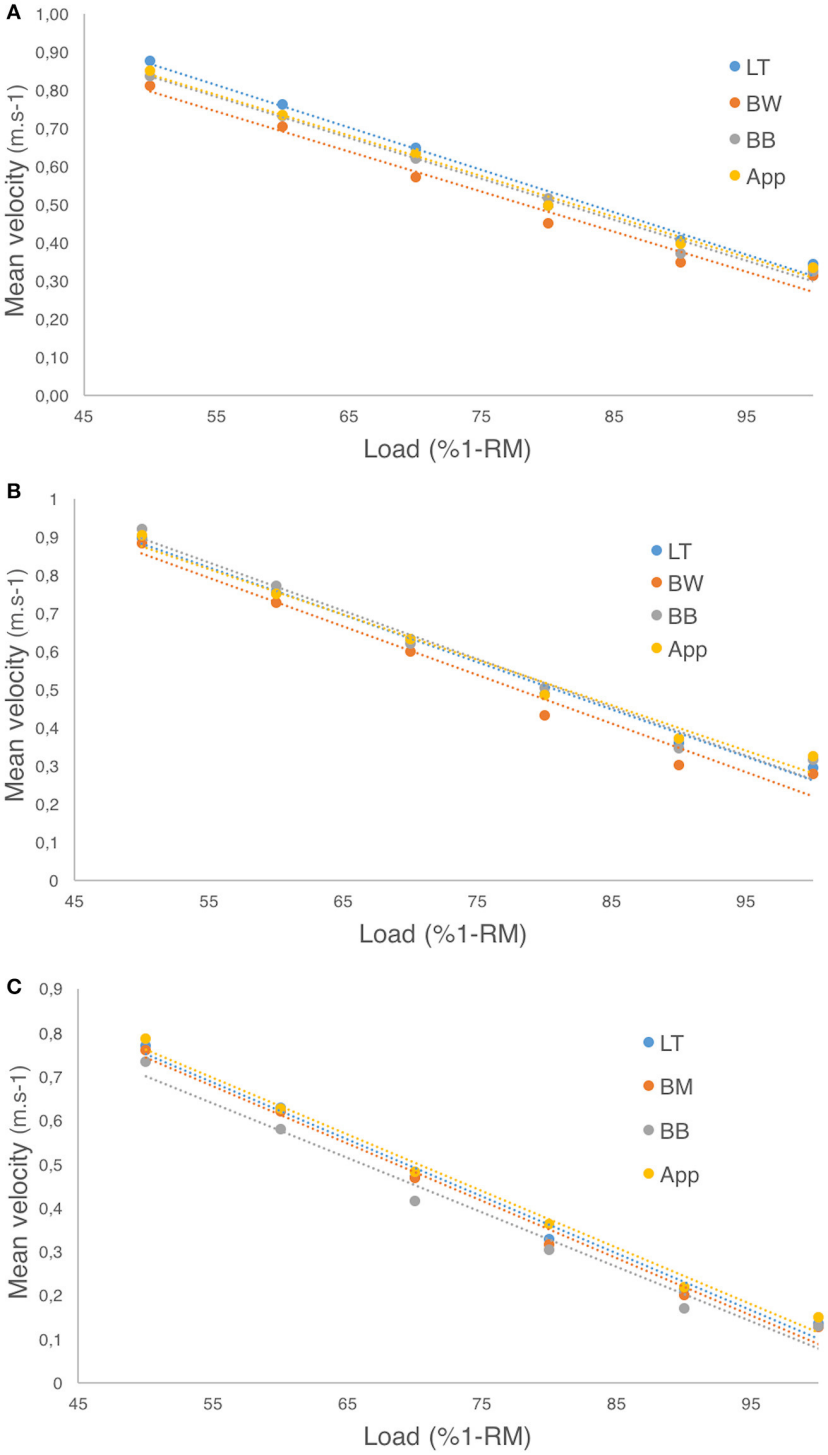
Load-velocity profiles computed from velocities obtained by each device for: **(A)** Hip-thrust; **(B)** Full-squat; **(C)** Bench-press. App: *PowerLift* app; BW = *Beast* sensor (wrist); BB = *Beast* sensor (barbell); LT = linear transducer.

When analyzing the individual load-velocity relationships of the three studied exercises using least-squares regression, no significant differences were observed in the slope, intercept or *R*^2^ values (*p* > 0.05) computed with the velocities measured with the LT, BW, BB, or *PowerLift* app as revealed by the one-way ANOVA. See Figure 4 for more details.

## Discussion

Both the *Beast* wearable device (in both of its configurations) and the *PowerLift* iOS app were found to have acceptable validity and reliability in comparison with a linear transducer (LT) for the measurement of barbell velocity in the full-squat, bench-press, and hip-thrust exercises. Specifically, bootstrapping analysis (*N* = 1,000) showed a very narrow confidence interval for Pearson's product-moment correlation coefficient (*r* = 0.94–0.98) for the three devices, highlighting the high level of agreement between BW, BB, *PowerLift*, and the LT for the measurement of barbell velocity. Moreover, although systematic biases were observed for velocities measured with BW, BB, and the app in the hip-thrust and bench-press exercises, the absolute difference was small in all cases (mean difference = −0.01 to 0.06 m•s^−1^) and, furthermore, the analysis of the regression lines of the Bland-Altman plots showed that differences between the novel devices and the gold standard were homogeneous across all the ranges of velocities measured (*R*^2^ = 0.001–0.03). These results are in line with previous research that showed very similar validity, reliability, and accuracy scores for the measurement of barbell velocity in the bench-press exercise using the *PowerLift* app (Balsalobre-Fernández et al., 2017) and confirms the suitability of high-speed video analysis to assess different activities such as jumping, sprinting, running, or lifting (Balsalobre-Fernández et al., 2015, 2016a; Romero-Franco et al., 2016; Sañudo et al., 2016). To the best of our knowledge, no previous studies have analyzed the *Beast* sensor for the measurement of movement velocity in resistance exercises; however, other wearable devices designed for the same purpose were recently studied (Balsalobre-Fernández et al., 2016b; Banyard et al., 2017). Specifically, the PUSH wearable device also showed high levels of validity and reliability in comparison with a linear transducer (*r* > 0.9) for the measurement of movement velocity (Balsalobre-Fernández et al., 2016b). However, results in our study showed that the *Beast* sensor, both attached to the barbell or to the wristband, has superior values of correlation and accuracy in comparison with a linear transducer working at 1 kHz. While the correlation between the PUSH band and a 1 kHz linear transducer was good for the measurement of back-squat (*r* = 0.86) in the mentioned study, this value is remarkably lower than those obtained both with BW and BB in our study for the same exercise (*r* = 0.960–0.983), meaning that the *Beast* sensor seems to be better associated with the measures obtained with a 1 kHz LT. Moreover, although both devices showed homogeneous differences across the whole range of velocities analyzed in comparison with the LT (as revealed by the Bland-Altman plots), the accuracy of the *Beast* sensor was also superior that the one observed with the PUSH band (0.01, 0.06, and 0.11 m•s^−1^ of mean difference with respect to LT for BB, BW, and PUSH band respectively). These differences could be in part because each wearable is designed to be placed in different body parts. While the *Beast* sensor is designed to be placed in a wristband or directly attached to the barbell, the PUSH band is meant to be placed just below the elbow of the subject. Therefore, the *Beast* sensor is much closer to the barbell than the PUSH band, which could provide better measures in comparison with a LT. However, to better compare these two wearables for the measurement of barbell velocity, more studies should be conducted measuring the exact same repetitions with respect to the same LT.

Another main result in our study was the comparison of the load-velocity profiles derived from the velocities measured with BW, BB, the *PowerLift* app, and the LT. The measurement of the load-velocity profiles (using a first-order linear regression fit) is of great interest since it describes the ability of the subject to produce velocity at loads of increasing intensity and it is used to estimate the 1-RM and each of its percentages with high accuracy without the need to conduct an actual 1-RM test (Jidovtseff et al., 2011; Picerno et al., 2016; Muñoz-López et al., 2017). Several studies have analyzed the load-velocity profiles on different exercises such as full-squat (Conceição et al., 2016), bench-press (Gonzalez-Badillo and Sánchez-Medina, 2010) or pull-up (Muñoz-López et al., 2017) and it was showed, in all cases, that the load (in terms of %1-RM) is highly correlated with the velocity at which each load is moved. Therefore, the analysis of the load-velocity profiles is getting an increasing interest in strength and conditioning and is the basis of the recent interest on the so-called velocity-based resistance training (Gonzalez-Badillo and Sánchez-Medina, 2010; González-Badillo et al., 2015; Muñoz-López et al., 2017). In this sense, we compared the slopes and y-intercepts of the load-velocity profiles computed with the velocities of BW, BB, the *PowerLift* app, and the LT to test if similar values were obtained. One way ANOVA showed no significant differences between BW, BB, or the *PowerLift* app and the LT (*p* > 0.05) for the three exercises analyzed. Moreover, the four regression lines are practically overlapped when they are plotted in the same graph (see Figure 4). Therefore, both the *Beast* sensor (in the wrist or in the barbell) and the *PowerLift* app are showed to provide accurate estimations of the load-velocity profiles of the subject and, consequently, can be used to estimate the 1-RM of the subjects.

It is worth noting that, while BB, BW, and the app had similar values of validity, reliability and accuracy for the measurement of barbell velocity with respect to the LT, the *Beast* sensor provided slightly better results in all tests when it was attached to the barbell in comparison with wearing it on the wrist. Therefore, although the *Beast* sensor is categorized as a wearable device, our results suggest that it not should be worn in the wrist, but attached to the barbell in order to get the more accurate results.

In conclusion, the *Beast* sensor (both in the wrist or attached to the barbell) and the *PowerLift* app were showed to be highly, valid, reliable, and accurate for the measurement of barbell velocity in the full-squat, bench-press, and hip-thrust exercises in comparison with a linear transducer. Moreover, these devices were proven to be highly suitable for the analysis of the load-velocity profiles in the mentioned exercises. These results could have potential interest for strength and conditioning coaches who wish to monitor movement velocity in the full-squat, bench-press, and hip-thrust exercises.

## Author contributions

I confirm that all authors have significantly contributed to this manuscript. The specific tasks developed by each researcher were as follows: CB, DM, MM, and SJ: design of the experiment. DM, MM, EB, and IA: data collection. CB, SJ, and MM: writing of the manuscript. CB: statistical analyses. All authors: revision of the manuscript.

### Conflict of interest statement

The first author of the article (CB) is the developer of the app mentioned. To guarantee the independency of the data collection, the data from the app were obtained by an independent researcher (IA), who has no personal or professional relationship with CB, and is not related to the app in any way. The other three authors who were in charge of the data collection as reported in the author contribution statement registered data from the other three devices analyzed in this study.
